# MED3/343: Networked Learner Support in Continuing Medical Education

**DOI:** 10.2196/jmir.1.suppl1.e46

**Published:** 1999-09-19

**Authors:** L Fox, E Dolman, D Hornby, P Lane, A O'Rourke, C Roberts, T Roscoe

**Affiliations:** ^1^University of Sheffield Community Sciences CentreSheffieldUK

**Keywords:** Education, Medical, Graduate, Information technology, Networked Professional Development, Networked Learner Support

## Abstract

**Introduction:**

This paper reports data from the second year of the WISDOM research project. This project has established a framework for the delivery of continuing medical education and professional development using information and communication technologies, with a particular focus on networked professional development (NPD) for clinical governance.

**Methods:**

An action research project.

**Results:**

The association of a web site providing resource materials (tutorials, electronic links and other information) with networking technologies, such as e-mail discussion groups and newsgroups, has been demonstrated to be effective in delivering curricula in informatics, evidence-based practice and reflective practice. The virtual classroom may be adapted to serve a number of objectives, and can be adapted to operate as a virtual conference and as a mechanism for research data collection methods, such as the Delphi methodology. The project's findings indicate that there is a need for a coherent policy of networked learner support (NLS) in such virtual classrooms. NLS includes:

- support on the development of adult-learning and reflection skills in participants
- active facilitation of learning within an adult-learning model
- mechanisms for assessment which reflect adult learning approaches (for example, a portfolio approach or personal learning plan)
- a mechanism for mentoring participants to enable them to achieve their learning objectives

**Conclusion:**

It is argued that NPD and NLS must be further researched if information and communication technologies are to be fully exploited as delivery mechanisms in professional development.

